# Crystal structure of 2-meth­oxy-2-[(4-meth­oxy­phen­yl)sulfan­yl]-1-phenyl­ethanone

**DOI:** 10.1107/S2056989015014565

**Published:** 2015-08-15

**Authors:** Ignez Caracelli, Paulo R. Olivato, Henrique J. Traesel, Jéssica Valença, Daniel N. S. Rodrigues, Edward R. T. Tiekink

**Affiliations:** a Departmento de Física, Universidade Federal de São Carlos, 13565-905 São Carlos, SP, Brazil; b Instituto de Química, Universidade de São Paulo, 05508-000 São Paulo, SP, Brazil; c Department of Chemistry, University of Malaya, 50603 Kuala Lumpur, Malaysia

**Keywords:** crystal structure, C—H⋯O inter­actions, β-thio­carbon­yl, conformation

## Abstract

In the title β-thio­carbonyl compound, C_16_H_16_O_3_S, the adjacent meth­oxy and carbonyl O atoms are synperiplanar [the O—C—C—O torsion angle is 19.8 (4)°] and are separated by 2.582 (3) Å. The dihedral angle between the rings is 40.11 (16)°, and the meth­oxy group is coplanar with the benzene ring to which it is connected [the C—C—O—C torsion angle is 179.1 (3)°]. The most notable feature of the crystal packing is the formation of methine and methyl C—H⋯O(carbon­yl) inter­actions that lead to a supra­molecular chain with a zigzag topology along the *c* axis. Chains pack with no specific inter­molecular inter­actions between them.

## Related literature   

For background to the present structural study, see: Vinhato *et al.* (2013 ▸); Zukerman-Schpector *et al.* (2008 ▸, 2015 ▸); Olivato *et al.* (2013 ▸); Distefano *et al.* (1996 ▸). For the structure of the methyl derivative, see: Zukerman-Schpector *et al.* (2015 ▸). For synthetic procedures, see: Ali & McDermott (2002 ▸); Zoretic & Soja (1976 ▸).
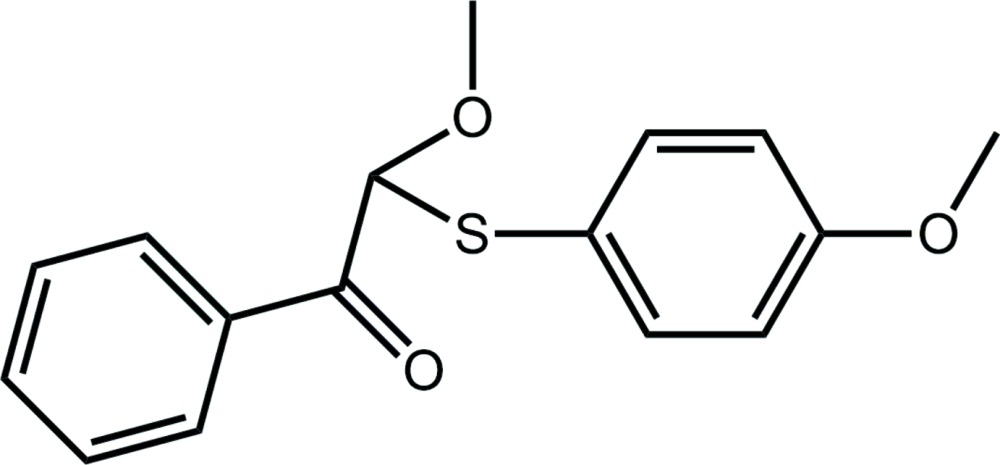


## Experimental   

### Crystal data  

C_16_H_16_O_3_S
*M* *_r_* = 288.35
Orthorhombic, 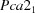

*a* = 18.769 (3) Å
*b* = 7.643 (1) Å
*c* = 10.0578 (16) Å
*V* = 1442.8 (4) Å^3^
*Z* = 4
Mo *K*α radiation
μ = 0.23 mm^−1^
*T* = 296 K
0.37 × 0.16 × 0.09 mm

### Data collection  

Bruker APEXII CCD diffractometer
Absorption correction: multi-scan (*SADABS*; Sheldrick, 1996 ▸) *T*_min_ = 0.618, *T*_max_ = 0.745
6725 measured reflections
1935 independent reflections
1627 reflections with *I* > 2σ(*I*)
*R*_int_ = 0.026

### Refinement  

*R*[*F* ^2^ > 2σ(*F* ^2^)] = 0.031
*wR*(*F* ^2^) = 0.076
*S* = 1.04
1935 reflections
183 parameters
1 restraint
H-atom parameters constrained
Δρ_max_ = 0.12 e Å^−3^
Δρ_min_ = −0.14 e Å^−3^
Absolute structure: Flack *x* determined using 418 quotients [(*I* ^+^)−(*I* ^−^)]/[(*I* ^+^)+(*I* ^−^)] (Parsons *et al.*, 2013 ▸)
Absolute structure parameter: 0.09 (4)

### 

Data collection: *APEX2* (Bruker, 2009 ▸); cell refinement: *SAINT* (Bruker, 2009 ▸); data reduction: *SAINT* (Bruker, 2009 ▸); program(s) used to solve structure: *SIR* (Burla *et al.*, 2015 ▸); program(s) used to refine structure: *SHELXL2014* (Sheldrick, 2015 ▸); molecular graphics: *ORTEP-3 for Windows* (Farrugia, 2012 ▸) and *DIAMOND* (Brandenburg, 2006 ▸); software used to prepare material for publication: *Marvinsketch* (ChemAxon, 2010 ▸) and *publCIF* (Westrip, 2010 ▸).

## Supplementary Material

Crystal structure: contains datablock(s) I, New_Global_Publ_Block. DOI: 10.1107/S2056989015014565/hg5455sup1.cif

Structure factors: contains datablock(s) I. DOI: 10.1107/S2056989015014565/hg5455Isup2.hkl

Supporting information file. DOI: 10.1107/S2056989015014565/hg5455Isup3.cml

**Fig. 1.**
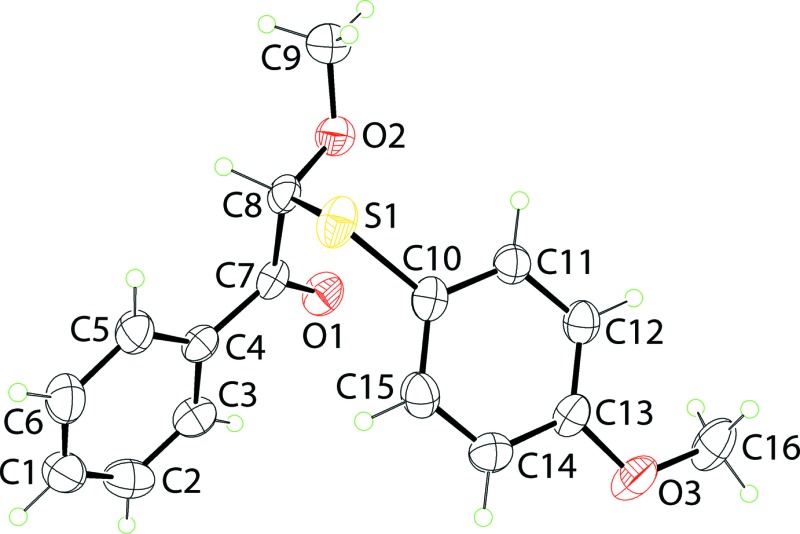
The molecular structure of the title compound showing the atom-labelling scheme and displacement ellipsoids at the 35% probability level.

. DOI: 10.1107/S2056989015014565/hg5455fig1.tif
The mol­ecular structure of the title compound showing the atom-labelling scheme and displacement ellipsoids at the 35% probability level.

**Fig. 2.**
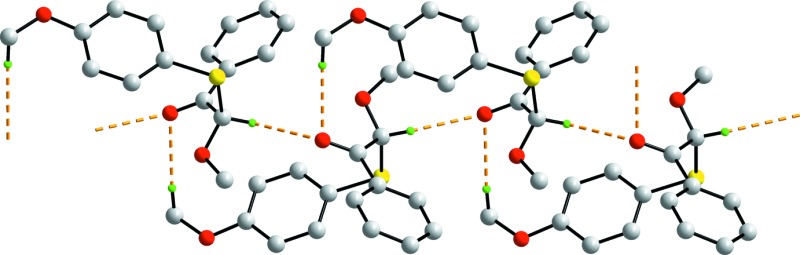
The supramolecular chain in the title compound sustained by C—H···O interactions shown as orange dashed lines. Hydrogen atoms not participating in C—H···O interactions have been omitted for reasons of clarity.

**Fig. 3.**
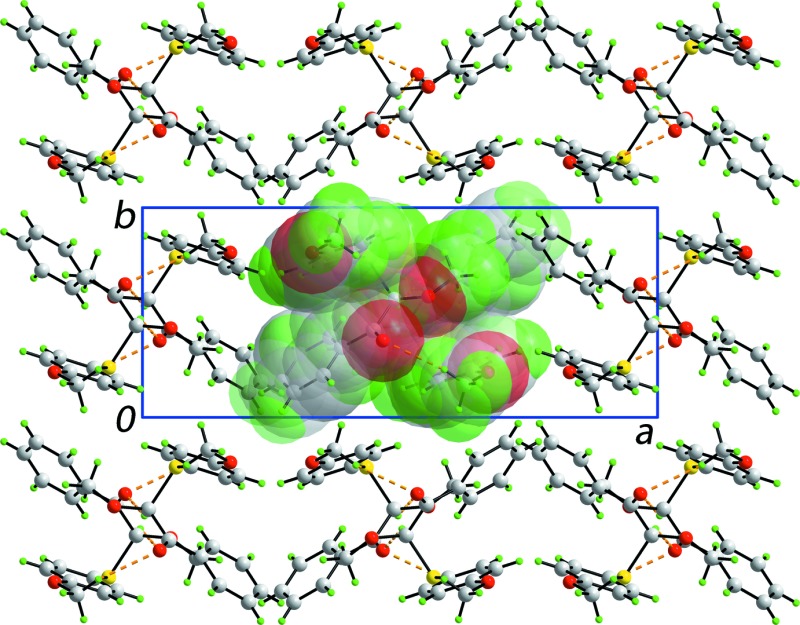
Unit-cell contents of the title compound shown in projection down the c axis. Intermolecular C—H···O interactions are shown as orange dashed lines. One supramolecular chain has been highlighted in space-filling mode.

. DOI: 10.1107/S2056989015014565/hg5455fig2.tif
The supra­molecular chain in the title compound sustained by C—H⋯O inter­actions shown as orange dashed lines. Hydrogen atoms not participating in C—H⋯O inter­actions have been omitted for reasons of clarity.

c . DOI: 10.1107/S2056989015014565/hg5455fig3.tif
Unit-cell contents of the title compound shown in projection down the *c* axis. Inter­molecular C—H⋯O inter­actions are shown as orange dashed lines. One supra­molecular chain has been highlighted in space-filling mode.

CCDC reference: 1416521

Additional supporting information:  crystallographic information; 3D view; checkCIF report

## Figures and Tables

**Table 1:** Hydrogen-bond geometry (, )

*D*H*A*	*D*H	H*A*	*D* *A*	*D*H*A*
C8H8O1^i^	0.98	2.54	3.406(5)	147
C16H16*C*O1^ii^	0.96	2.47	3.421(5)	170

Symmetry codes: (i) 
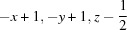
; (ii) 
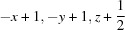
.

## supplementary crystallographic information

### S1. Introduction

As part of our on-going research into the conformational and electronic inter­actions of β-thio-carbonyl, β-bis-thio-carbonyl and β-thio-β-oxa-carbonyl compounds, *e.g*. N,N-di­ethyl-2-[(4'-substituted)phenyl­thio]­acetamides, 1-methyl-3-phenyl­sulfonyl-2-piperidone, 3,3-bis­[(4'-substituted)phenyl­sulfanyl]-1-methyl-2-piperidones, 2-alkyl­thio-2-alkyl­sulfinyl-aceto­phenones, 2-alkyl­thio-2-phenyl­sulfonyl-aceto­phenones, 2-alkyl­sulfinyl-2-alkyl­sulfonyl-aceto­phenones and 2-meth­oxy-2-[(4'-methyl­phenyl)­sulfanyl]-1-phenyl­ethan-1-one, utilizing spectroscopic, theoretical and X-ray diffraction methods (Distefano *et al.*, 1996; Zukerman-Schpector *et al.*, 2008; Olivato *et al.*, 2013; Vinhato *et al.*, 2013; Zukerman-Schpector *et al.*, 2015) the title compound was synthesized and its crystal structure determined.

### S2.1. Synthesis and crystallization

4'-Meth­oxy­thio­phenol (5.0 g, 36 mmol) was reacted with bromine (1.1 ml, 20 mmol) in di­chloro­methane (250 ml) on an hydrated silica gel support (25 g of SiO_2_ and 12 ml of water) to give 4'-meth­oxy­phenyl di­sulfide (4.0 g, yield = 80%). A white solid was obtained after filtration and evaporation without further purification (Ali & McDermott, 2002). A solution of 2-meth­oxy aceto­phenone (0.80 ml, 5.81 mmol, Sigma-Aldrich) in THF (20 ml), was added drop-wise to a cooled (195 K) solution of diiso­propyl­amine (0.90 ml, 6.39 mmol) and butyl­lithium (4.30 ml, 5.81 mmol) in THF (30 ml). After 30 minutes, a solution of 4'-meth­oxy­phenyl di­sulfide (1.780 g, 6.39 mmol) with hexa­methyl­phospho­ramide (HMPA) (1.0 ml, 5.81 mmol) dissolved in THF (20 ml) was added drop-wise to the enolate solution (Zoretic & Soja, 1976). After stirring for 3 h, water (50 ml) was added at room temperature and extraction with di­chloro­methane was performed. The organic layer was then treated with saturated solution of ammonium chloride until neutral pH, and then dried over anhydrous magnesium sulfate. A brown oil was obtained after evaporation of the solvent. Purification through flash chromatography with toluene was used to remove the non-polar rea­ctant (di­sulfide) then acetone to give a mixture of both aceto­phenones (product and rea­ctant). Crystallization was performed by vapour diffusion of *n*-hexane into a chloro­form solution held at 283 K to give pure product (0.3 g, yield = 40%). Suitable crystals for X-ray diffraction were obtained by same pathway; m.pt: 393.5-394.2 K. IR (cm^-1^): ν(C=O) 1695 (CCl_4_). ^1^H NMR (CDCl_3_, 500 MHz, δ p.p.m.): 3.68 (s, 3*H*), 3.78 (s, 3*H*), 5.76 (s, 1*H*), 6.80–6.82 (m, 2*H*), 7.24–7.26 (m, 2*H*), 7.43–7.46 (m, 2*H*), 7.56–7.59 (m, 1*H*), 7.94–7.95 (m, 2*H*). Analysis for C_16_H_16_O_3_S: calculated (%): C 66.64, H 5.59; found (%): C 66.52, H 5.53. High-Resolution MS calculated (M^+^): 288.0820; found (M^+^): 288.0821.

### S2.2. Refinement

Carbon-bound H-atoms were placed in calculated positions (C—H = 0.93–0.98 Å) and were included in the refinement in the riding model approximation, with *U*_iso_(H) = 1.2–1.5*U*_eq_(C).

### Crystal data

**Table:**

C_16_H_16_O_3_S	*D*_x_ = 1.327 Mg m^−^^3^
*M**_r_* = 288.35	Mo *K*α radiation, λ = 0.71073 Å
Orthorhombic, *P**c**a*2_1_	Cell parameters from 2745 reflections
*a* = 18.769 (3) Å	θ = 2.2–25.1°
*b* = 7.643 (1) Å	µ = 0.23 mm^−^^1^
*c* = 10.0578 (16) Å	*T* = 296 K
*V* = 1442.8 (4) Å^3^	Irregular, colourless
*Z* = 4	0.37 × 0.16 × 0.09 mm
*F*(000) = 608	

### Data collection

**Table:**

Bruker APEXII CCD diffractometer	1627 reflections with *I* > 2σ(*I*)
φ and ω scans	*R*_int_ = 0.026
Absorption correction: multi-scan (*SADABS*; Sheldrick, 1996)	θ_max_ = 25.4°, θ_min_ = 2.2°
*T*_min_ = 0.618, *T*_max_ = 0.745	*h* = −22→22
6725 measured reflections	*k* = −8→9
1935 independent reflections	*l* = −7→12

### Refinement

**Table:**

Refinement on *F*^2^	Hydrogen site location: inferred from neighbouring sites
Least-squares matrix: full	H-atom parameters constrained
*R*[*F*^2^ > 2σ(*F*^2^)] = 0.031	*w* = 1/[σ^2^(*F*_o_^2^) + (0.0337*P*)^2^ + 0.2623*P*] where *P* = (*F*_o_^2^ + 2*F*_c_^2^)/3
*wR*(*F*^2^) = 0.076	(Δ/σ)_max_ < 0.001
*S* = 1.03	Δρ_max_ = 0.12 e Å^−^^3^
1935 reflections	Δρ_min_ = −0.14 e Å^−^^3^
183 parameters	Absolute structure: Flack *x* determined using 418 quotients [(*I*^+^)-(*I*^-^)]/[(*I*^+^)+(*I*^-^)] (Parsons *et al*., 2013)
1 restraint	Absolute structure parameter: 0.09 (4)

### Special details

**Table:**

Geometry. All e.s.d.'s (except the e.s.d. in the dihedral angle between two l.s. planes) are estimated using the full covariance matrix. The cell e.s.d.'s are taken into account individually in the estimation of e.s.d.'s in distances, angles and torsion angles; correlations between e.s.d.'s in cell parameters are only used when they are defined by crystal symmetry. An approximate (isotropic) treatment of cell e.s.d.'s is used for estimating e.s.d.'s involving l.s. planes.

### Fractional atomic coordinates and isotropic or equivalent isotropic displacement parameters (Å^2^)

**Table:**

	*x*	*y*	*z*	*U*_iso_*/*U*_eq_	
S1	0.56536 (5)	0.23724 (10)	0.74255 (12)	0.0585 (3)	
O1	0.53351 (13)	0.6345 (3)	0.9109 (3)	0.0608 (6)	
O2	0.44516 (10)	0.4243 (3)	0.7959 (3)	0.0588 (6)	
O3	0.67048 (13)	0.2149 (3)	1.2961 (3)	0.0702 (8)	
C1	0.7294 (2)	0.8300 (5)	0.6168 (5)	0.0670 (11)	
H1	0.7687	0.8842	0.5784	0.080*	
C2	0.70618 (18)	0.8808 (4)	0.7391 (5)	0.0707 (11)	
H2	0.7294	0.9710	0.7834	0.085*	
C3	0.64887 (17)	0.8002 (4)	0.7975 (4)	0.0566 (9)	
H3	0.6338	0.8355	0.8814	0.068*	
C4	0.61332 (15)	0.6666 (4)	0.7325 (4)	0.0441 (7)	
C5	0.63593 (19)	0.6186 (5)	0.6074 (4)	0.0587 (9)	
H5	0.6117	0.5320	0.5608	0.070*	
C6	0.6949 (2)	0.6995 (5)	0.5506 (4)	0.0696 (11)	
H6	0.7108	0.6644	0.4672	0.083*	
C7	0.55159 (15)	0.5857 (4)	0.8007 (3)	0.0439 (7)	
C8	0.51146 (15)	0.4382 (3)	0.7343 (4)	0.0455 (7)	
H8	0.5041	0.4685	0.6407	0.055*	
C9	0.3949 (2)	0.3296 (5)	0.7185 (4)	0.0718 (12)	
H9A	0.4129	0.2143	0.7012	0.108*	
H9B	0.3871	0.3894	0.6358	0.108*	
H9C	0.3507	0.3213	0.7663	0.108*	
C10	0.59396 (18)	0.2334 (4)	0.9103 (4)	0.0448 (8)	
C11	0.55035 (17)	0.1718 (4)	1.0103 (4)	0.0474 (8)	
H11	0.5045	0.1348	0.9897	0.057*	
C12	0.57361 (17)	0.1641 (4)	1.1405 (4)	0.0493 (9)	
H12	0.5434	0.1230	1.2068	0.059*	
C13	0.64116 (18)	0.2170 (4)	1.1714 (4)	0.0491 (9)	
C14	0.68553 (18)	0.2783 (4)	1.0714 (4)	0.0610 (10)	
H14	0.7316	0.3143	1.0919	0.073*	
C15	0.66189 (18)	0.2860 (4)	0.9429 (4)	0.0550 (9)	
H15	0.6921	0.3273	0.8767	0.066*	
C16	0.6275 (2)	0.1561 (6)	1.4024 (4)	0.0749 (11)	
H16A	0.6538	0.1639	1.4841	0.112*	
H16B	0.6138	0.0367	1.3871	0.112*	
H16C	0.5856	0.2277	1.4083	0.112*	

### Atomic displacement parameters (Å^2^)

**Table:**

	*U*^11^	*U*^22^	*U*^33^	*U*^12^	*U*^13^	*U*^23^
S1	0.0873 (6)	0.0513 (4)	0.0370 (5)	0.0083 (4)	−0.0003 (5)	−0.0065 (5)
O1	0.0732 (14)	0.0705 (15)	0.0387 (16)	0.0031 (13)	0.0019 (12)	−0.0129 (13)
O2	0.0561 (13)	0.0760 (15)	0.0444 (16)	−0.0089 (11)	0.0015 (13)	−0.0003 (13)
O3	0.0628 (15)	0.0992 (19)	0.0486 (18)	0.0162 (14)	−0.0127 (15)	0.0049 (15)
C1	0.056 (2)	0.067 (2)	0.078 (4)	−0.0075 (19)	−0.006 (2)	0.015 (2)
C2	0.061 (2)	0.061 (2)	0.090 (4)	−0.0085 (17)	−0.010 (3)	−0.010 (3)
C3	0.061 (2)	0.0537 (18)	0.055 (3)	0.0065 (17)	−0.007 (2)	−0.0090 (18)
C4	0.0491 (16)	0.0415 (14)	0.042 (2)	0.0075 (13)	−0.0069 (16)	−0.0003 (17)
C5	0.073 (2)	0.064 (2)	0.039 (2)	−0.0115 (18)	−0.0002 (19)	−0.0035 (18)
C6	0.075 (2)	0.083 (3)	0.051 (3)	−0.001 (2)	0.009 (2)	0.008 (2)
C7	0.0561 (17)	0.0455 (16)	0.030 (2)	0.0080 (14)	−0.0052 (17)	0.0020 (15)
C8	0.0552 (17)	0.0530 (16)	0.0282 (17)	−0.0006 (14)	−0.0009 (16)	0.0002 (18)
C9	0.070 (2)	0.081 (2)	0.065 (3)	−0.020 (2)	−0.009 (2)	0.002 (2)
C10	0.0572 (19)	0.0356 (16)	0.041 (2)	0.0100 (14)	0.0040 (16)	−0.0001 (15)
C11	0.0507 (18)	0.0478 (18)	0.044 (2)	−0.0021 (14)	0.0007 (16)	0.0018 (16)
C12	0.0536 (19)	0.0496 (19)	0.045 (2)	0.0071 (15)	0.0068 (16)	0.0041 (16)
C13	0.053 (2)	0.055 (2)	0.040 (2)	0.0139 (16)	−0.0016 (18)	0.0033 (16)
C14	0.0455 (18)	0.075 (2)	0.063 (3)	0.0020 (17)	−0.004 (2)	0.008 (2)
C15	0.055 (2)	0.060 (2)	0.050 (3)	0.0023 (17)	0.0125 (19)	0.0059 (18)
C16	0.083 (2)	0.103 (3)	0.039 (3)	0.027 (3)	−0.001 (2)	0.005 (2)

### Geometric parameters (Å, º)

**Table:**

S1—C10	1.771 (4)	C7—C8	1.511 (4)
S1—C8	1.841 (3)	C8—H8	0.9800
O1—C7	1.217 (4)	C9—H9A	0.9600
O2—C8	1.394 (3)	C9—H9B	0.9600
O2—C9	1.421 (4)	C9—H9C	0.9600
O3—C13	1.370 (4)	C10—C15	1.376 (5)
O3—C16	1.413 (5)	C10—C11	1.380 (5)
C1—C2	1.361 (6)	C11—C12	1.381 (5)
C1—C6	1.363 (6)	C11—H11	0.9300
C1—H1	0.9300	C12—C13	1.367 (5)
C2—C3	1.372 (5)	C12—H12	0.9300
C2—H2	0.9300	C13—C14	1.387 (5)
C3—C4	1.384 (4)	C14—C15	1.368 (6)
C3—H3	0.9300	C14—H14	0.9300
C4—C5	1.377 (5)	C15—H15	0.9300
C4—C7	1.482 (4)	C16—H16A	0.9600
C5—C6	1.390 (5)	C16—H16B	0.9600
C5—H5	0.9300	C16—H16C	0.9600
C6—H6	0.9300		
			
C10—S1—C8	102.90 (15)	O2—C9—H9A	109.5
C8—O2—C9	112.8 (3)	O2—C9—H9B	109.5
C13—O3—C16	117.8 (3)	H9A—C9—H9B	109.5
C2—C1—C6	119.8 (4)	O2—C9—H9C	109.5
C2—C1—H1	120.1	H9A—C9—H9C	109.5
C6—C1—H1	120.1	H9B—C9—H9C	109.5
C1—C2—C3	120.7 (4)	C15—C10—C11	118.4 (3)
C1—C2—H2	119.7	C15—C10—S1	120.2 (3)
C3—C2—H2	119.7	C11—C10—S1	121.4 (3)
C2—C3—C4	120.4 (4)	C10—C11—C12	121.2 (3)
C2—C3—H3	119.8	C10—C11—H11	119.4
C4—C3—H3	119.8	C12—C11—H11	119.4
C5—C4—C3	118.6 (3)	C13—C12—C11	119.8 (3)
C5—C4—C7	123.6 (3)	C13—C12—H12	120.1
C3—C4—C7	117.8 (3)	C11—C12—H12	120.1
C4—C5—C6	120.2 (4)	C12—C13—O3	125.3 (3)
C4—C5—H5	119.9	C12—C13—C14	119.5 (4)
C6—C5—H5	119.9	O3—C13—C14	115.2 (3)
C1—C6—C5	120.2 (4)	C15—C14—C13	120.3 (4)
C1—C6—H6	119.9	C15—C14—H14	119.8
C5—C6—H6	119.9	C13—C14—H14	119.8
O1—C7—C4	120.8 (3)	C14—C15—C10	120.8 (3)
O1—C7—C8	119.4 (3)	C14—C15—H15	119.6
C4—C7—C8	119.8 (3)	C10—C15—H15	119.6
O2—C8—C7	107.8 (2)	O3—C16—H16A	109.5
O2—C8—S1	114.0 (2)	O3—C16—H16B	109.5
C7—C8—S1	109.2 (2)	H16A—C16—H16B	109.5
O2—C8—H8	108.6	O3—C16—H16C	109.5
C7—C8—H8	108.6	H16A—C16—H16C	109.5
S1—C8—H8	108.6	H16B—C16—H16C	109.5
			
C6—C1—C2—C3	0.9 (6)	C4—C7—C8—S1	74.1 (3)
C1—C2—C3—C4	−0.5 (5)	C10—S1—C8—O2	−74.0 (3)
C2—C3—C4—C5	−1.1 (5)	C10—S1—C8—C7	46.6 (3)
C2—C3—C4—C7	−179.6 (3)	C8—S1—C10—C15	−101.2 (3)
C3—C4—C5—C6	2.2 (5)	C8—S1—C10—C11	81.4 (3)
C7—C4—C5—C6	−179.3 (3)	C15—C10—C11—C12	0.6 (4)
C2—C1—C6—C5	0.2 (6)	S1—C10—C11—C12	178.0 (2)
C4—C5—C6—C1	−1.9 (6)	C10—C11—C12—C13	−0.5 (5)
C5—C4—C7—O1	−179.3 (3)	C11—C12—C13—O3	−179.7 (3)
C3—C4—C7—O1	−0.8 (4)	C11—C12—C13—C14	0.2 (5)
C5—C4—C7—C8	2.1 (4)	C16—O3—C13—C12	−1.1 (5)
C3—C4—C7—C8	−179.4 (3)	C16—O3—C13—C14	179.1 (3)
C9—O2—C8—C7	161.9 (3)	C12—C13—C14—C15	0.1 (5)
C9—O2—C8—S1	−76.7 (3)	O3—C13—C14—C15	179.9 (3)
O1—C7—C8—O2	19.8 (4)	C13—C14—C15—C10	0.0 (5)
C4—C7—C8—O2	−161.5 (2)	C11—C10—C15—C14	−0.3 (5)
O1—C7—C8—S1	−104.5 (3)	S1—C10—C15—C14	−177.8 (3)

### Hydrogen-bond geometry (Å, º)

**Table:**

*D*—H···*A*	*D*—H	H···*A*	*D*···*A*	*D*—H···*A*
C8—H8···O1^i^	0.98	2.54	3.406 (5)	147
C16—H16*C*···O1^ii^	0.96	2.47	3.421 (5)	170

Symmetry codes: (i) −*x*+1, −*y*+1, *z*−1/2; (ii) −*x*+1, −*y*+1, *z*+1/2.
