# Supplementary figures and images for: Crystal structure of 2-meth­oxy-2-[(4-meth­oxy­phen­yl)sulfan­yl]-1-phenyl­ethanone
Source: Acta Crystallogr E Crystallogr Commun. 2015 Aug 15;71(Pt 9):o657–8. doi: 10.1107/S2056989015014565 (PMC4555440; doi:10.1107/S2056989015014565)

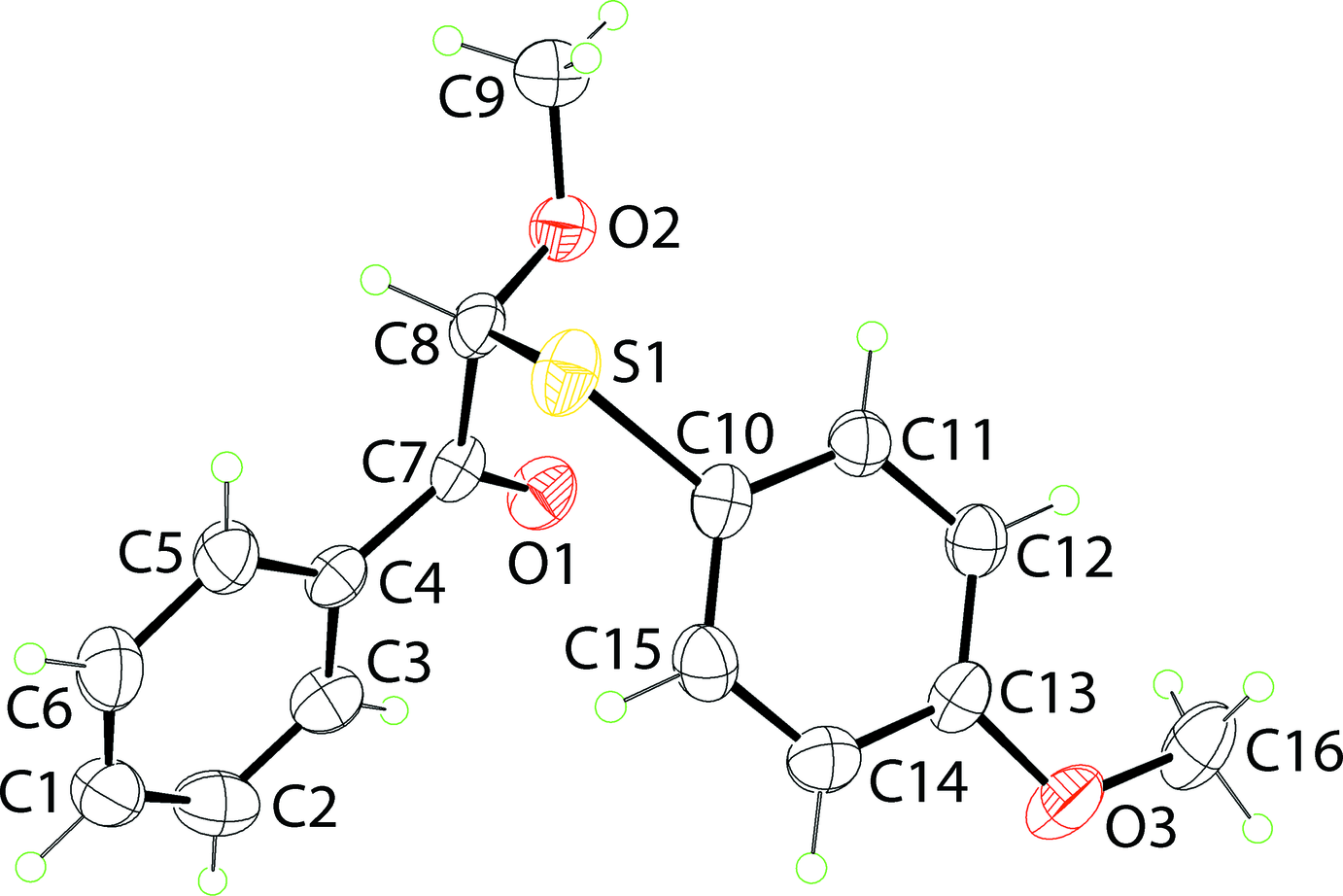

Supplement: Supplementary file 4 [file e-71-0o657-fig1.tif]

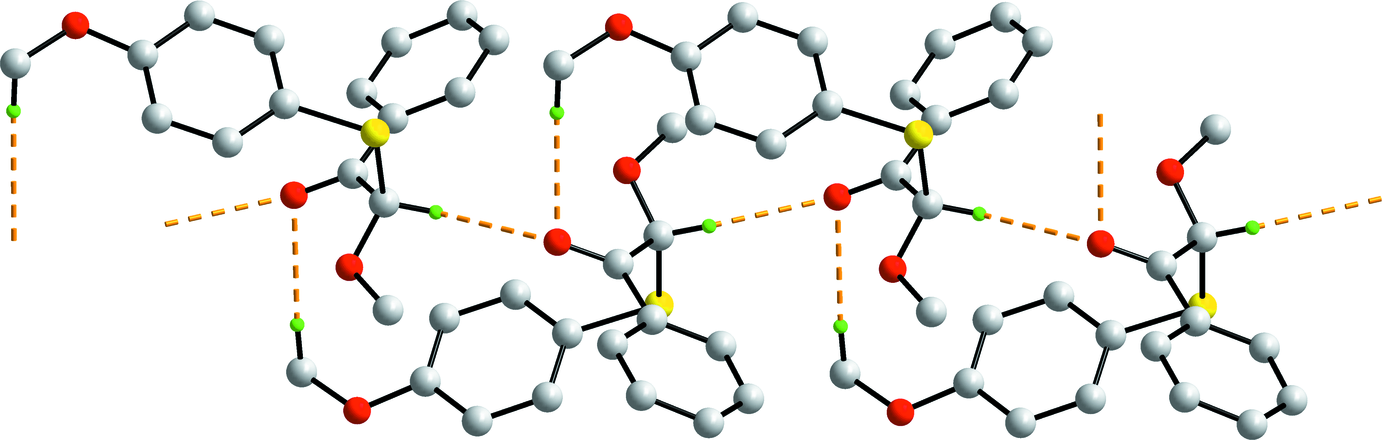

Supplement: Supplementary file 5 [file e-71-0o657-fig2.tif]

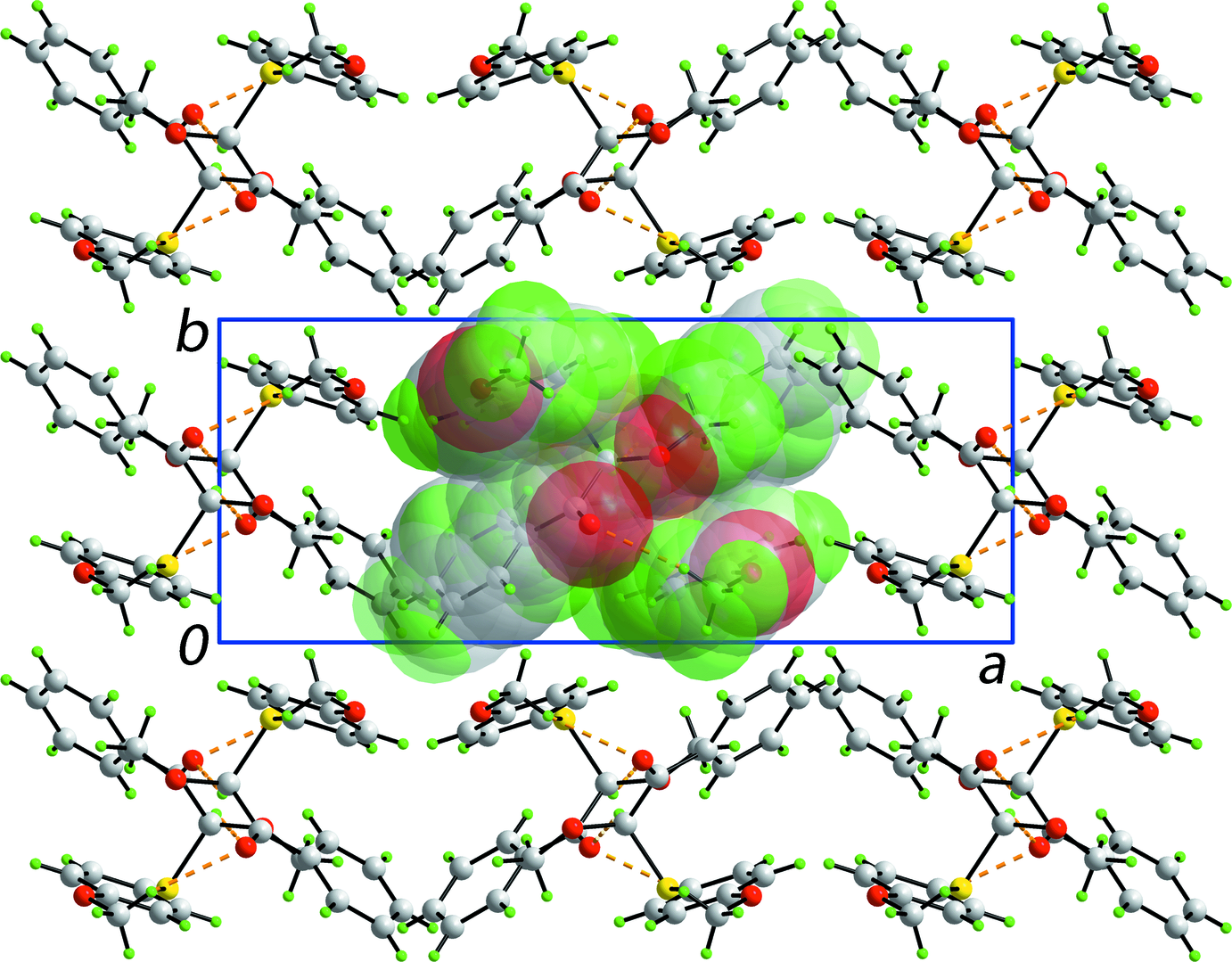

Supplement: Supplementary file 6 [file e-71-0o657-fig3.tif]
